# Early Identification of Resuscitated Patients with a Significant Coronary Disease in Out-of-Hospital Cardiac Arrest Survivors without ST-Segment Elevation

**DOI:** 10.3390/jcm10235688

**Published:** 2021-12-02

**Authors:** Chun-Song Youn, Hahn Yi, Youn-Jung Kim, Hwan Song, Namkug Kim, Won-Young Kim

**Affiliations:** 1 Department of Emergency Medicine, Seoul St. Mary’s Hospital, College of Medicine, The Catholic University of Korea, Seoul 06591, Korea; ycs1005@catholic.ac.kr (C.-S.Y.); cmcmdsong@gmail.com (H.S.); 2 Asan Medical Center, Asan Institute for Life Sciences, Seoul 05505, Korea; hahn.yi@gmail.com; 3 Department of Emergency Medicine, Asan Medical Center, University of Ulsan College of Medicine, Seoul 05505, Korea; yjkim.em@gmail.com; 4 Department of Convergence Medicine, Asan Medical Center, University of Ulsan College of Medicine, Seoul 05505, Korea

**Keywords:** out-of-hospital cardiac arrest, coronary angiography, machine learning, coronary artery disease

## Abstract

This study aimed to develop a machine learning (ML)-based model for identifying patients who had a significant coronary artery disease among out-of-hospital cardiac arrest (OHCA) survivors without ST-segment elevation (STE). This multicenter observational study used data from the Korean Hypothermia Network prospective registry (KORHN-PRO) gathered between October 2015 and December 2018. We used information available before targeted temperature management (TTM) as predictor variables, and the primary outcome was a significant coronary artery lesion in coronary angiography (CAG). Among 1373 OHCA patients treated with TTM, 331 patients without STE who underwent CAG were enrolled. Among them, 127 patients (38.4%) had a significant coronary artery lesion. Four ML algorithms, namely regularized logistic regression (RLR), random forest classifier (RF), CatBoost classifier (CBC), and voting classifier (VC), were used with data collected before CAG. The VC model showed the highest accuracy for predicting significant lesions (area under the curve of 0.751). Eight variables (older age, male, initial shockable rhythm, shorter total collapse duration, higher glucose and creatinine, and lower pH and lactate) were significant to ML models. These results showed that ML models may be useful in developing early predictive tools for identifying high-risk patients with a significant stenosis in CAG.

## 1. Introduction

Coronary artery disease is the main cause of out-of-hospital cardiac arrest (OHCA) [1]. Immediate reperfusion of the culprit coronary lesion is proposed to improve outcome in OHCA survivors [2]. Current international guidelines recommend that coronary angiography (CAG) be performed emergently for all cardiac arrest patients with suspected cardiac cause of arrest and ST-segment elevation (STE) on electrocardiogram (ECG) [3]. However, in OHCA patients without STE, early CAG is suggested for selected patients, but guidelines do not provide specific characteristics of patients who may benefit from immediate CAG [4]. Thus, the main challenge is to identify the best candidates for CAG among resuscitated cardiac arrest patients without STE. Previous research focused on the selection of CAG candidates who will recover with good neurologic outcomes. The benefit of CAG is associated primarily with providing percutaneous coronary intervention (PCI), and therefore, identifying patients without STE who have a significant coronary occlusion is crucial. OHCA survivors without STE do not always have obstructive coronary artery disease, and therefore, identifying these patients is complicated. Clinical findings such as chest pain are often lacking, and troponin levels can be increased in resuscitated patients even without acute coronary causes. Therefore, the decision for CAG should consider multiple factors, including previous medical history, symptoms before the arrest, initial cardiac arrest rhythm, laboratory results, and ECG patterns after the return of spontaneous survival (ROSC).

Newer computational methods, namely machine learning (ML), may allow more accurate prediction than risk assessment tools developed using standard methods. Targeted ML algorithms triggered by patient data have been increasingly developed as clinical decision support tools in various diseases, including sepsis, gastrointestinal bleeding, and cardiac arrest [5,6,7]. Given the complexity and time dependency of OHCA survivors without STE, ML-based methods are expected to provide a good foundation for selecting tools for identifying the best candidates for early CAG among OHCA survivors without STE. This study aimed to develop an ML-based model for identifying patients with a significant coronary artery disease among OHCA survivors without STE.

## 2. Materials and Methods

### 2.1. Study Design

This multicenter prospective observational study used data from the Korean Hypothermia Network prospective registry (KORHN-PRO) 1.0 gathered between October 2015 and December 2018. KORHN is a multicenter clinical research consortium for targeted temperature management (TTM) in South Korea. Twenty-two academic hospitals participated in KORHN-PRO. The study included an informed consent form approved by all participating hospitals, and the study was registered at the International Clinical Trials Registry Platform (NCT02827422). Written informed consent was obtained from all patients′ legal surrogates.

### 2.2. Population and Variables

The inclusion criteria of KORHN-PRO were as follows: OHCA regardless of etiology of cardiac arrest, age older than 18 years, unconsciousness (Glasgow Coma Scale score < 8) after ROSC, and treatment with TTM. The exclusion criteria were as follows: active intra-cranial bleeding, acute stroke, known limitations in therapy and a do-not-attempt resuscitation order, known prearrest cerebral performance category (CPC) 3 or 4, known disease making 6-month survival unlikely, and body temperature < 30 °C on admission. Data were entered into a web-based electronic database registry using a standardized registry form. Each participating hospital had a designated research coordinator responsible for ensuring data accuracy.

For the present trial, we excluded patients with obvious non-cardiac causes and STE on the initial ECG from KORHN-PRO. This study used 37 independent and one dependent variable described below from the registry: age, sex, comorbidities (previous arrest, previous acute myocardial infarction, previous angina, previous arrhythmia, chronic heart failure, previous transient ischemic attack (TIA) or stroke, hypertension, diabetes mellitus, pulmonary disease, neurologic disease other than cerebrovascular accident (CVA), chronic kidney disease, malignancy, previous PCI, previous coronary artery bypass grafting, and previous ischemic heart disease), family history (cardiac arrest, stroke, angina and myocardial infarction, arrhythmia, unknown), resuscitation variables (initial shockable rhythm, witnessed arrest, bystander cardiopulmonary resuscitation, time from collapse to ROSC, total epinephrine dose), post-ROSC variables (immediate ECG findings and laboratory findings), and extracorporeal life support. OHCA survivors were defined as OHCA patients who had a return of spontaneous circulation and were admitted to the hospital for management of post-cardiac arrest care including TTM. The outcome variable of this work was whether OHCA survivors without STE had a significant coronary artery lesions. Significant coronary artery disease was defined by invasive CAG as >50% stenosis of the left main stem or >70% stenosis in a major coronary vessel.

### 2.3. ML Algorithms

Through automated ML procedures, four ML algorithms (regularized logistic regression (RLR) [8], random forest (RF) [9], CatBoost classifier (CBC) [10], and voting classifier (VC) [11], which combined the other three models by 1:1:1) were selected to predict patients needing CAG among OHCA survivors without STE. For curated data, 29 out of 37 independent variables are categorical variables, and the dependent variable is binary. Linearity between the independent variables and the dependent variable cannot be assumed.

Logistic regression (LR) is a statistical model that uses the logistic function as a link function to explain the relationship between independent and dependent variables. In the field of ML, an arbitrary constant is added to the cost function of LR and used as a model for classifying unseen data. When learning a model with training data, the constant helps to optimize between the bias and variance by preventing overfitting. LR with this constraint is called RLR, and the coefficients of explanatory variables in the optimized model represent the feature importance of each variable.

RF is known as an algorithm that can create an optimal model for prediction or classification in a short learning time from a tabular data structure in which continuous and categorical variables are mixed. In particular, it is possible to prevent overfitting by extracting part of the data through sampling and generating a number of mutually independent decision trees using only some variables that greatly reduce the cost function, predicting the outcome through majority voting and evaluating the importance of the independent features. These are the reasons we adopted RF in this study.

In our data, as most independent variables are categorical variables and most of the categorical independent variables are binary, it is essential for algorithms to handle binary categorical variables well. RF may have difficultly generating optimized models if many binary categorical variables are included because it creates a model in a deep tree structure with variables having only one splitting point. As the name suggests, CatBoost can effectively handle categorical variables through sequentially encoding categorical variables or combining highly correlated categorical variables. In addition, because CatBoost uses the boosting algorithm, it is possible to generate an optimized decision tree model in a way different from that of RF.

The three algorithms with different strengths and weaknesses create each optimized model. Finally, the voting algorithm was used to combine the prediction probabilities of the models with several voting methods to form an ensemble model.

Multiclass classification by splitting a minor outcome group into two subgroups was conducted to enhance the performance of the models. Additionally, analyses were carried out by feature selection by permutation [12] or the dimensional compression method with principal component analysis [13] and uniform manifold approximation and projection [14]. However, these trials made no model performance improvement. A stratified five-fold cross-validation method was applied to avoid overfitted model generation, and realistic model performance was evaluated in a clinical environment. A random search method [15] was used for optimizing hyperparameters so that the Cohen’s kappa of the RLR, RF, and CBC models had the highest values. For model evaluations, the predicted ratio of patients with significant lesions among OHCA patients without STE, the area under the receiver operating characteristic (AUROC), log loss, sensitivity, specificity, positive predictive value (PPV), negative predictive value (NPV), F1-score (the harmonic average of sensitivity and PPV), Cohen’s kappa, and net reclassification improvement (NRI) were used. The performance of the models was evaluated as a function of the number of sample data used to train the models and the number of variables included in descending order of relative feature importance (RFI) in the model. Lastly, the VC model’s explainability for global feature importance was presented using Shapely values. Python [16] and its extension packages, such as NumPy 1.20.3 [17], scikit-learn 0.24.2 [18], pandas 1.2.0 [19], SciPy 1.6.1 [20], matplotlib 3.3.2 [21], CatBoost 0.24.4 [10], shap 0.39.0 [22], TPOT 0.11.7 [23], and PyCaret 2.3.1 [24], were used for the ML analyses.

### 2.4. Statistical Methods

Continuous variables are presented as the means with the standard deviation or as median values with interquartile ranges (IQRs). Categorical variables are presented as frequencies and percentages. For patient characteristics and comparisons between groups (significant lesion and without significant lesion), we used Student′s t-test for normally distributed continuous variables and the Mann–Whitney U test for nonparametric cases. Fisher′s exact test was used for cases of low-frequency categorical variables, and the chi-square test was used otherwise. *p*-values ≤ 0.05 indicated statistical significance.

In our dataset, nine variables had missing values, and Little’s missing completely at random test [25] was run on the dataset to confirm that all variables with missing values in our data were missing completely at random. In the process of data imputation, to increase statistical reliability, the predictive mean matching algorithm of the multivariate imputation by chained equations (MICE) package was used to produce 30 different datasets [26]. A combination of 30 imputed datasets and 5-fold cross-validation allowed stable mean values and 95% confidence intervals (CIs) of the evaluation metrics to be computed. Statistical analysis was performed using R version 4.1.0 beta [27] and its packages such as the MICE 3.13.0 [26].

## 3. Results

### 3.1. Baseline Statistics

During the study period, a total of 1373 OHCA patients (>18 years) treated with TTM were enrolled in KORHN-PRO (Figure S1). Among them, 521 patients with obvious non-cardiac causes of arrest, 11 patients lacking ECG data, four patients with no information of outcome, and 163 patients with STE were excluded. Further, 343 patients dropped out due to no CAG within 2 weeks. Thus, 331 patients without STE who underwent CAG were ultimately included in this study.

Among them, 127 patients (38.4%) had a significant coronary artery lesion. Table 1 presents the baseline characteristics by classifying patients according to significant coronary lesion or no lesion. Several variables (age, male, previous acute myocardial infarction, diabetes mellitus, chronic kidney disease, ST-segment depression, glucose, creatinine, and extracorporeal life support) were statistically different between the two groups.

### 3.2. Model Performance and Validation

**Figure 1:**
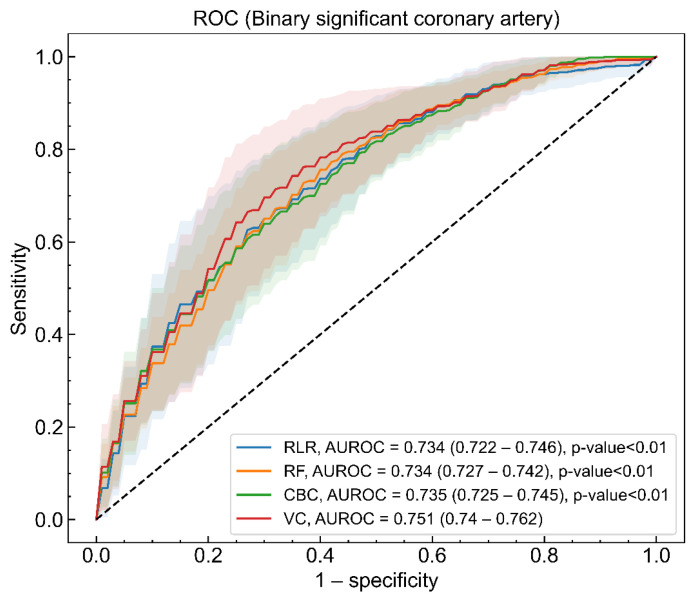
Area under the receiver operating characteristic (AUROC) of regularized logistic regression (RLR, blue), random forest (RF, orange), CatBoost classifier (CBC, green), and voting classifier (VC, red) models for binary significant coronary artery lesion. The solid lines and shaded regions represent the mean and standard deviation of AUROCs for models, respectively. The mean value of AUROC and 95% confidence interval (CI) for the four models and the statistical test result, indicating that the AUROC of the VC model is different from those of other models, are presented in the legend.

The four ML models were trained to identify patients who had a significant coronary artery disease among OHCA survivors without STE. The AUROC (95% CI) values of the trained RLR, RF, CBC, and VC models were 0.734 (0.722–0.746), 0.737 (0.729–0.745), 0.736 (0.726–0.746), and 0.751 (0.740–0.762), respectively (Figure 1). The AUROC of the VC model was statistically better than those of the RLR, RF, and CBC models at a significance level of 0.05. Figure S2 shows the sensitivity, specificity, PPV, and NPV of the four learning models measured in response to increments of thresholds from 0 to 1.

The mean and 95% CI of loss and metrics values were estimated with the 150 models made by a combination of 30 imputed datasets and a five-fold cross validation procedure, and the results are shown in Table 2. All four models had better performance than a random classifier at the absolute values of log loss, considering the ratio of the dependent variable labels (38.4%) [28]. The VC model was superior to the other three models based on all kinds of metrics from AUROC to NRI. The fact that the lower 95% CI of NRI for the VC model was larger than zero revealed that the VC model was only better than the RLR model [29].

**Figure 2:**
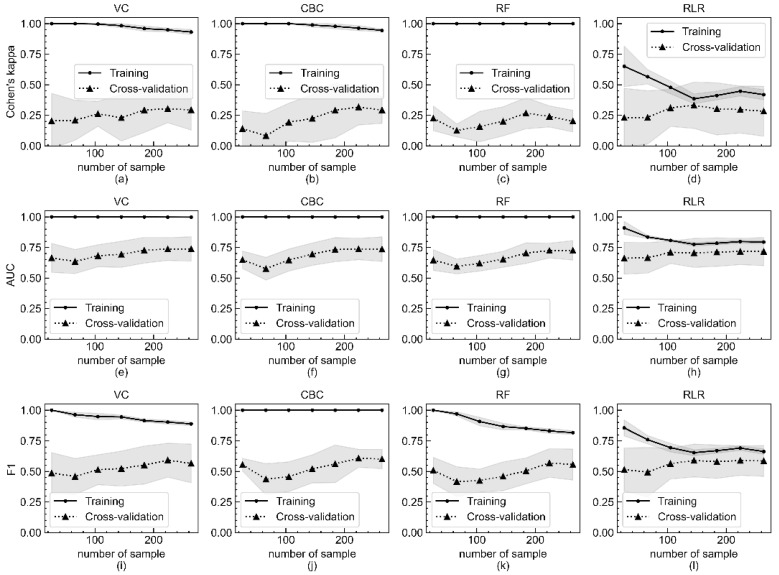
Metric score changes of Cohen’s kappa, AUROC, and F1-score of four models according to number of sample data included in training and evaluation by five-fold cross validation. Changes of Cohen’s kappa of (**a**) VC, (**b**) CBC, (**c**) RF, and (**d**) RLR models according to number of sample data included in training and evaluation by five-fold cross validation. Changes of AUROC of (**e**) VC, (**f**) CBC, (**g**) RF, and (**h**) RLR models. Changes of F1-score of (**i**) VC, (**j**) CBC, (**k**) RF, and (**l**) RLR models. The *x*-axis is the number of sample data included in the experiment, and the *y*-axis is the calculated metric value. The mean metric values of the training set and the testing set are respectively represented as sold lines with filled circles and dotted lines with filled triangles. The shaded region indicates the standard deviation of all metrics.

The learning curves for Cohen’s kappa, AUROC, and F1-score of the VC, CBC, RF, and RLR models as a function of the number of sample data are shown in Figure 2. As the number of sample data increases, the scores of the model evaluated with the training set gradually decrease and then become flattened, and those of the model evaluated with the testing set increase and then reach a saturated state. Figure 2 implies that the performance of all four models can be enhanced when more sample data are collected.

**Figure 3:**
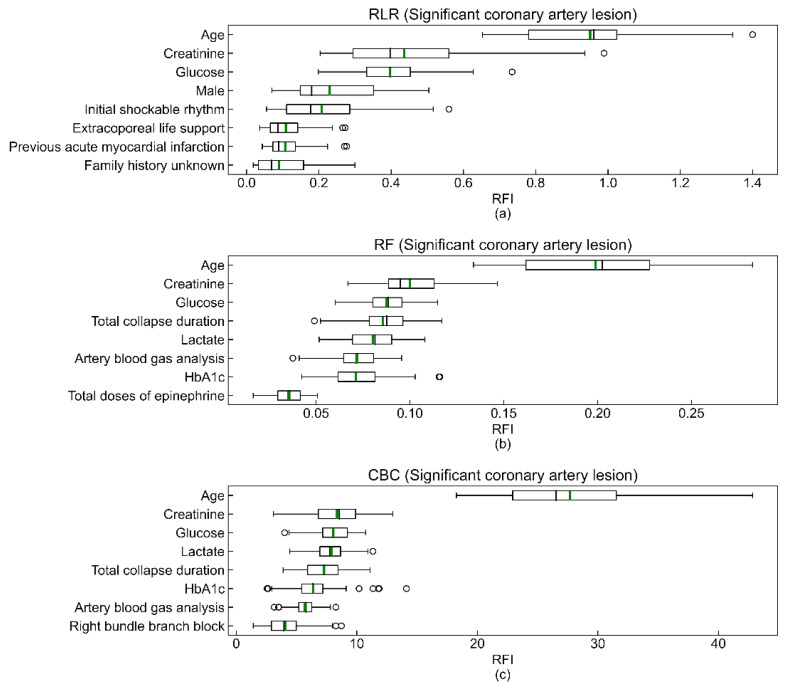
Top eight features of RLR (**a**), RF (**b**), and CBC (**c**) models. The box extends from the first quartile (Q1) to the third quartile (Q3), which is also called IQR. The black and green bars in the box are the median and the mean of the relative feature importance (RFI). The left whisker end is Q1-1.5IQR, and the right whisker end is Q3 + 1.5IQR. Lastly, the hollow circles outside of both whisker ends are the outliers of the coefficients of RFI. The total sums of all feature importance coefficients of the RF and CBC models are 1 and 100, respectively.

The coefficients of feature importance were respectively estimated by the (a) mean of coefficients of RLR models, (b) Gini impurity-based method of the RF model, and (c) prediction values changed algorithm of CBC models (Figure 3). Note that the values on the *x*-axis in Figure 3 are meaningful only as the relative importance between features for a model. The order of the Top 3 variables of feature importance (age, creatinine, and glucose) was consistent in the three models. In addition, the predictor variables shown to be statistically significant in Table 1 tend to have high feature importance coefficients.

**Figure 4:**
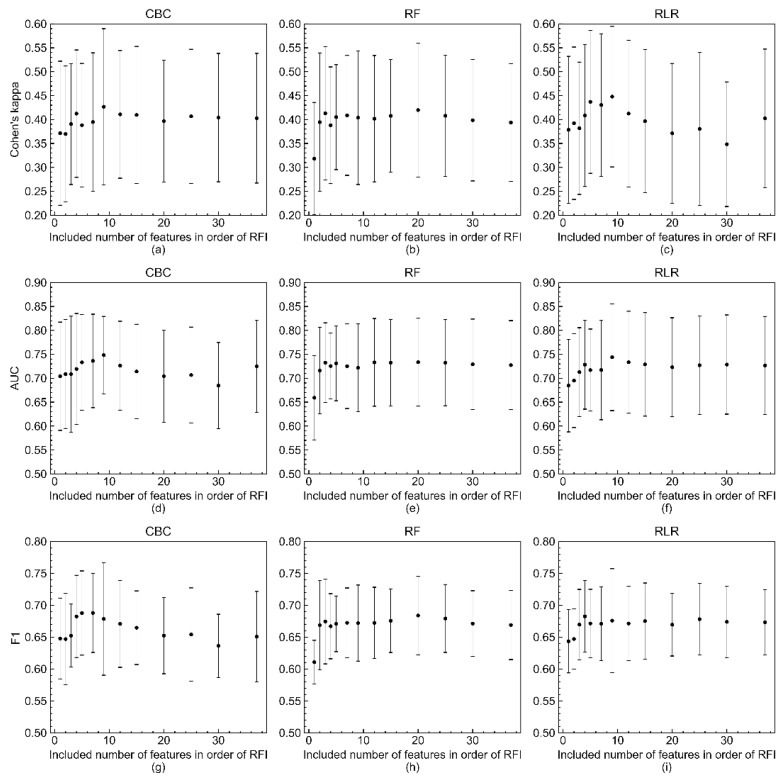
Changes of three metric scores (Cohen’s kappa, AUROC, and F1-score) of RLR, RF, and CBC models in response to number of included variables in order of RFI. Changes of Cohen’s kappa of (**a**) CBC, (**b**) RF, and (**c**) RLR models in response to number of included variables in order of RFI. Changes of AUROC of (**d**) CBC, (**e**) RF, and (**f**) RLR models. Changes of F1-score of (**g**) CBC, (**h**) RF, and (**i**) RLR models. The mean values and standard deviations of the three metric scores are represented by filled circles and bars with caps, respectively. The three metrics were calculated when the numbers of features were 1, 2, 3, 4, 5, 7, 9, 12, 15, 20, 25, 30, and 37 due to the time-consuming model training procedure and lack of computing resources.

Figure 4 shows the results of evaluating the model’s performance with three metrics—Cohen’s kappa, AUROC, and F1-score—according to the number of variables included in the model. The three metric scores were computed by increasing the number of predicting variables in descending order of feature importance coefficients. The number of variables and the imputed datasets included in this experiment were limited due to the lack of computing resources and time-consuming training procedure. In all three metrics, it appeared that the model built with only the top nine variables in terms of feature importance was not inferior to the model with all 37 predicting variables.

The global feature importance for the VC model, the best-performing model in this study, was calculated using Shapley values to reliably select important variables to identify patients with a significant coronary artery lesion. In Figure S3, the features that are higher on the vertical axis have a greater influence on predicting the outcome. When a value of a patient’s variable is located on the right side of the horizontal axis, the observed value contributes to increasing the probability of predicting that the patient will have a significant coronary artery lesion. More specifically, older age, male, initial shockable rhythm, shorter total collapse duration, higher glucose and creatinine, and lower pH and lactate predicted the higher probability of a positive outcome.

## 4. Discussion

In this study, we developed models based on ML algorithms to predict patients with a significant coronary artery lesion requiring early PCI through only basic patient information before admission. We found that the VC model showed the highest accuracy for predicting a significant lesion (AUROC of 0.751). We also found that eight easily captured variables (older age, male, initial shockable rhythm, shorter total collapse duration, higher glucose and creatinine, and lower pH and lactate) were significant.

Coronary artery disease is particularly prevalent in cardiac arrest patients with shockable rhythm: up to 96% of patients with STE on their post-resuscitation ECG and up to 42% for patients without STE [30,31]. Our study showed that 38.4% of patients had a significant coronary artery lesion. This is in agreement with recent studies that have found coronary occlusions in approximately 30% of patients without STE [32,33,34].

Considering that the benefit of CAG is associated primarily with providing PCI, identifying patients who have a significant coronary occlusion is crucial. Waldo et al. developed a risk prediction model for the presence of an acute coronary lesion among patients resuscitated from an arrest [35]. They found four variables— angina, congestive heart failure, shockable rhythm, and STE—and STE had the highest number of points. However, few studies regarding OHCA survivors without STE have focused on the selection of CAG candidates based on neurologic outcome [36,37,38,39]. Although selecting OHCA survivors without STE who will have good neurologic outcomes is important, the next step will be to identify which OHCA survivors have a significant coronary occlusion. Comparing immediate and delayed CAG in cardiac arrest patients with STE, recent randomized controlled trials, such as Coronary Angiography after Cardiac Arrest (COACT) and Angiography after Out-of-Hospital Cardiac Arrest without ST-Segment Elevation (TOMAHAWK), and a systematic review failed to show the superiority of immediate CAG over delayed CAG in improving the survival of resuscitated OHCA patients without STE [40,41]. Thus, the aim of our study was to develop an ML-based model for the early (within 24 h) identification of patients with a significant coronary artery lesion.

For now, except those with STE or cardiogenic shock, the specific subgroups that would benefit from early CAG remain unknown [2,3]. A study from the United States using the International Cardiac Arrest Registry developed a prediction model of CREST scores for circulatory-etiology death using data obtained at the time of intensive care unit admission in OHCA patients without STE. History of coronary artery disease, non-shockable rhythm, initial ejection fraction <30%, shock at the time of presentation, and total ischemic time >25 min accurately and pragmatically predicted the risk of circulatory-etiology death [42]. The American College of Cardiology Interventional Council suggested triaging OHCA survivors using 10 unfavorable resuscitation features, determined by consensus: unwitnessed arrest, initial rhythm non-ventricular fibrillation, no bystander cardiopulmonary resuscitation, >30 min from collapse to ROSC (time-to-ROSC), ongoing cardiopulmonary resuscitation, pH < 7.2, lactate > 7 mmol/L, age > 85 years, end-stage renal disease, and non-cardiac etiology [43]. Moreover, a very recent study by Harhash et al. aimed to identify resuscitated cardiac arrest patients with unfavorable clinical features for whom invasive procedures are unlikely to improve survival [36]. They found that the most powerful predictors of adverse outcome were age > 85 years, >30 min before return of spontaneous circulation, and initial non-shockable rhythm. Although these variables were for predicting poor outcome, some variables, such as age, duration of cardiopulmonary resuscitation, shockable rhythm, and pH in blood gas analysis, were the same as factors for predicting a significant coronary lesion from ML. Noh et al. investigated one prediction ML algorithm to select the high-risk group of patients with acute coronary syndrome requiring revascularization using data from 2344 patients [44]. The obtained prediction functions were relevant, showing an AUROC of 0.860 for the prediction of ACS requiring revascularization. They found the main obstacles to applying the ML techniques were missing data and uncertain labeling causing small numbers of available data.

The data that accumulate in a stressful emergency room are affected by several biases, including typos and discontinuous laboratory measurement. They have great impacts on ML studies using data made up of already sculpted tabular-type variables. However, it was possible to create better performing VC models that synthesized the effective RLR, RF, and CBC models that were complementary with advantages of different algorithms. The soft voting method, the use of the arithmetic mean of prediction probabilities from the RLR, RF, and CBC models, worked well in this study.

The aim of this research was to determine which variables greatly hint at the presence of significant coronary artery lesions. Feature importance determined using Shapley values considering all possible combinations of variables in the VC model can be considered the most reliable result. This provides guidelines to practitioners in emergency rooms on the need to carefully monitor patients with the following: older age, male, initial shockable rhythm, shorter total collapse duration, higher glucose and creatinine, and lower pH and lactate. This result can also be used to prioritize which variables in data to collect by investing limited emergency room resources.

As shown in Figure 4, eight to eleven independent variables of top feature importance of RLR, RF, and CBC showed good performance comparable to that of optimized models with 37 predicting variables. As mentioned earlier, this result suggests that better clinical outcomes might be produced if current limited resources are focused on compiling important variables. Figure 1, Figure 2, Figure 3 and Figure 4 depict large standard deviations of almost all metrics, which seem to be due to the insufficient number of sample data, not the inclusion of important variables, and the difficulty in selecting representative values of time-series attribute variables [45]. The AUROC values of the model, inferior to that with all 37 variables, trained with the complete dataset to build a model for rapid response under the current emergency room environment are presented in Figure S4. We also observed that the missing value issue, which Noh et al. noted in 2019, is a barrier that makes it difficult to employ ML algorithms in clinical practice [44].

This study has several limitations. There is an important concern about the population selected. KORHN-PRO did not have a solid indication of CAG except STE or cardiogenic shock. The selection of the patients who had undergone CAG was done by the treating physicians, which means there was already a high suspicion that these patients had ACS, creating selection bias. Other issues include the requirement of a computer implementation for the ML model and the limited power to detect other clinical characteristics that may be associated with a significant coronary artery disease due to the modest sample size. Moreover, to appropriately select patients that would benefit from CAG, thrombotic occlusion or culprit lesion was more useful than a significant coronary artery disease. Lastly, there may be valuable variables such as results of echocardiography not included in our registry. Therefore, our VC model is not complete enough to be used in the clinical field right now. Further larger studies with other variables will be needed.

## 5. Conclusions

ML models may be helpful early predictive tools for identifying high-risk patients with a significant stenosis in CAG. However, further investigations with large datasets including other variables are warranted to improve the prediction model, which can support clinicians’ decisions on early CAG in OHCA survivors without STE.

## Figures and Tables

**Table 1:** Baseline characteristics of study patients with and without significant lesion.

Variables	Significant Lesion (*n* = 127)	No Significant Lesion (*n* = 204)	*p*-Value	Missing Value Ratio (%)
Age, years	63.4 ± 11.93	52.7 ± 14.27	<0.001	0
Male sex	107 (84.3%)	149 (73.0%)	0.03	0
Comorbid disease				
Previous arrest	1 (0.79%)	3 (1.47%)	0.97	0
Previous acute myocardial infarction	20 (15.8%)	11 (5.39%)	0.003	0
Previous angina	11 (8.66%)	20 (9.80%)	0.88	0
Previous arrhythmia	7 (5.51%)	11 (5.39%)	0.84	0
Chronic heart failure	3 (2.36%)	7 (3.43%)	0.82	0
Previous transient ischemic attack (TIA) or stroke	8 (6.30%)	5 (2.45%)	0.14	0
Hypertension	55 (43.3%)	66 (32.4%)	0.06	0
Diabetes mellitus	36 (28.4%)	36 (17.7%)	0.03	0
Pulmonary disease	0 (0%)	2 (0.98%)	0.70	0
Neurologic disease other than cerebrovascular accident (CVA)	4 (3.15%)	3 (1.47%)	0.52	0
Chronic kidney disease	11 (8.66%)	4 (1.96%)	0.01	0
Malignancy	3 (2.36%)	13 (6.37%)	0.16	0
Previous percutaneous coronary intervention (PCI)	9 (7.09%)	7 (3.43%)	0.21	0
Previous coronary artery bypass grafting (CABG)	5 (3.94%)	2 (0.98%)	0.15	0
Previous ischemic heart disease	24 (18.9%)	17 (8.33%)	0.008	0
Family history of				
cardiac arrest	1 (0.790%)	4 (1.96%)	0.70	0
Angina or acute myocardial infarction (AMI)	5 (3.94%)	6 (2.94%)	0.86	0
CVA	1 (0.790%)	5 (2.45%)	0.50	0
Arrhythmia	0 (0%)	3 (1.47%)	0.44	0
Unknown	110 (86.6%)	169 (82.8%)	0.45	0
arrest characteristics				
Initial shockable rhythm	91 (71.7%)	148 (72.6%)	0.87	6.0
Witnessed	107 (84.3%)	165 (80.9%)	0.43	0.3
Bystander cardiopulmonary resuscitation (CPR)	83 (65.4%)	138 (67.7%)	0.94	1.2
Total collapse duration, min	21.0 (13.0–35.0)	23.0 (15.0–36.3)	0.38	0
Total doses of epinephrine, mg	2.44 ± 3.91	2.13 ± 4.00	0.52	10.6
Immediate electrocardiogram (ECG) findings				
ST-segment depression	69 (54.3%)	141 (69.1%)	0.009	0
Right bundle branch block	95 (74.8%)	170 (83.3%)	0.08	0
Non-specific ST-segment or T wave changes	93 (73.2%)	129 (63.2%)	0.08	0
Normal ST-segment and T wave	112 (88.2%)	164 (80.4%)	0.09	0
Immediate laboratory findings				
Artery blood gas analysis, pH	7.13 ± 0.181	7.17 ± 0.184	0.07	4.5
Lactate, mg/dL	8.66 ± 4.830	8.41 ± 4.781	0.65	4.5
Glucose, mg/dL	286.0 ± 107.39	258.8 ± 93.18	0.02	1.2
Creatinine, mg/dL	1.83 ± 1.734	1.37 ± 0.887	0.006	0.3
Glycated hemoglobin (HbA1c, %)	6.14 ± 0.894	5.91 ± 1.094	0.11	41.4
Extracorporeal life support	17 (13.4%)	11 (5.39%)	0.02	0

Values are presented as mean ± standard deviation, median (IQR (interquartile range)), or number (percentage), as appropriate.

**Table 2:** Loss and metrics of four models.

Model	Actual	RLR (95% CI)	RF (95% CI)	CBC (95% CI)	VC (95% CI)
Predicted	0.383	0.439 (0.430–0.448)	0.540 (0.526–0.554)	0.295 (0.289–0.301)	0.437 (0.403–0.470)
AUROC	-	0.731 (0.719–0.744)	0.737 (0.729–0.746)	0.734 (0.724–0.744)	0.751 (0.740–0.762)
Log loss	-	0.206 (0.202–0.211)	0.207 (0.205–0.208)	0.229 (0.227–0.232)	0.204 (0.202–0.207)
Sensitivity	-	0.758 (0.738–0.779)	0.777 (0.760–0.793)	0.797 (0.778–0.817)	0.788 (0.767–0.809)
Specificity	-	0.672 (0.646–0.698)	0.661 (0.638–0.683)	0.630 (0.605–0.655)	0.682 (0.660–0.705)
Positive predictive value (PPV)	-	0.610 (0.595–0.626)	0.604 (0.590–0.618)	0.590 (0.575–0.606)	0.620 (0.607–0.633)
Negative predictive value (NPV)	-	0.829 (0.819–0.839)	0.832 (0.825–0.840)	0.843 (0.833–0.853)	0.850 (0.840–0.861)
F1-score	-	0.685 (0.671–0.700)	0.696 (0.685–0.707)	0.681 (0.666–0.696)	0.710 (0.697–0.724)
Kappa	-	0.412 (0.393–0.432)	0.416 (0.399–0.434)	0.402 (0.381–0.423)	0.448 (0.428–0.467)
Net reclassification improvement (NRI)	-	-	0.007 (−0.005–0.019)	−0.003 (−0.020–0.014)	0.040 (0.032–0.048)

NRI is a quantifying index of how much better the new model is than the old one.

## Data Availability

The data presented in this study are available on request from the corresponding author. The data are not publicly available due to the policy of Korea.

## Supplementary Materials

The following are available online at https://www.mdpi.com/article/10.3390/jcm10235688/s1: Figure S1: Study Patient Flow Diagram. ECG, electrocardiogram; OHCA, out-of-hospital cardiac arrest; STE, ST-segment elevation; TTM, targeted temperature management. Figure S2: Four metrics (sensitivity (blue), specificity (orange), PPV (green), NPV (red)) for RLR (a), RF (b), CBC (c), and VC (d) models in response to thresholds, ranging from 0 to 1; PPV, positive predictive value; NPV, negative predictive value; RLR, regularized logistic regression; RF, random forest; CBC, CatBoost classifier; VC, voting classifier. Figure S3: Feature importance of voting classifier model was estimated by Shapley value. The *x*-axis is the voting classifier model’s SHAP values, which are the contributions of each variable value to predicting the model outcome as label 1. When it is positive, the possibility of the existence of a significant lesion is increased, and vice versa. The variables higher on the *y*-axis have more influence on predicting the outcome in the model. The colored dots are sample data from the training set, and the red and blue dots indicate, respectively, high and low values in the range of each variable. ABGA, arterial blood gas analysis; HbA1c, glycated hemoglobin; ROSC, return of spontaneous survival. Figure S4: ROC and AUROC of four models (RLR (blue), RF (orange), CBC (green), VC (red)) trained on complete set excluding nine variables with missing values. AUROC, area under the receiver operating characteristic; RLR, regularized logistic regression; RF, random forest; CBC, CatBoost classifier; VC, voting classifier. Table S1: The definition and collection form of the variables in Korean Hypothermia Network prospective registry (KORHN-PRO).

## References

[1] Spaulding C.M., Joly L.-M., Rosenberg A., Monchi M., Weber S.N., Dhainaut J.-F.A., Carli P. (1997). Immediate coronary angiography in survivors of out-of-hospital cardiac arrest. N. Engl. J. Med..

[2] Yannopoulos D., Bartos J.A., Aufderheide T.P., Callaway C.W., Deo R., Garcia S., Halperin H.R., Kern K.B., Kudenchuk P.J., Neumar R.W. (2019). The Evolving Role of the Cardiac Catheterization Laboratory in the Management of Patients with Out-of-Hospital Cardiac Arrest: A Scientific Statement from the American Heart Association. Circulation.

[3] Panchal A.R., Bartos J.A., Cabañas J.G., Donnino M.W., Drennan I.R., Hirsch K.G., Kudenchuk P.J., Kurz M.C., Lavonas E.J., Morley P.T. (2020). Part 3: Adult Basic and Advanced Life Support: 2020 American Heart Association Guidelines for Cardiopulmonary Resuscitation and Emergency Cardiovascular Care. Circulation.

[4] Nolan J.P., Soar J., Cariou A., Cronberg T., Moulaert V.R., Deakin C.D., Bottiger B.W., Friberg H., Sunde K., Sandroni C. (2015). European resuscitation council and European society of intensive care medicine 2015 guidelines for post-resuscitation care. Intensive Care Med..

[5] Liu R., Greenstein J.L., Granite S.J., Fackler J.C., Bembea M.M., Sarma S.V., Winslow R.L. (2019). Data-driven discovery of a novel sepsis pre-shock state predicts impending septic shock in the ICU. Sci. Rep..

[6] Seo D.-W., Yi H., Park B., Kim Y.-J., Jung D.H., Woo I., Sohn C.H., Ko B.S., Kim N., Kim W.Y. (2020). Prediction of Adverse Events in Stable Non-Variceal Gastrointestinal Bleeding Using Machine Learning. J. Clin. Med..

[7] Seo D.-W., Yi H., Bae H.-J., Kim Y.-J., Sohn C.-H., Ahn S., Lim K.-S., Kim N., Kim W.-Y. (2021). Prediction of Neurologically Intact Survival in Cardiac Arrest Patients without Pre-Hospital Return of Spontaneous Circulation: Machine Learning Approach. J. Clin. Med..

[8] Salehi F., Abbasi E., Hassibi B. (2019). The Impact of Regularization on High-dimensional Logistic Regression. arXiv.

[9] Breiman L. (2001). Random forests. Mach. Learn..

[10] Prokhorenkova L., Gusev G., Vorobev A., Dorogush A.V., Gulin A. CatBoost: Unbiased boosting with categorical features. Proceedings of the Advances in Neural Information Processing Systems.

[11] Maclin R., Opitz D. (2011). Popular Ensemble Methods: An Empirical Study. arXiv.

[12] Kursa M.B., Rudnicki W.R. (2010). Feature selection with the Boruta package. J. Stat. Softw..

[13] Cadima J.F., Jolliffe I.T. (2001). Variable selection and the interpretation of principal subspaces. J. Agric. Biol. Environ. Stat..

[14] McInnes L., Healy J., Melville J. (2018). Umap: Uniform manifold approximation and projection for dimension reduction. arXiv.

[15] Bergstra J., Bengio Y. (2012). Random search for hyper-parameter optimization. J. Mach. Learn. Res..

[16] Van Rossum G., Drake F.L. (1995). Python Tutorial.

[17] Oliphant T.E. (2006). A Guide to NumPy.

[18] Pedregosa F., Varoquaux G., Gramfort A., Michel V., Thirion B., Grisel O., Blondel M., Prettenhofer P., Weiss R., Dubourg V. (2011). Scikit-learn: Machine learning in Python. J. Mach. Learn. Res..

[19] McKinney W. (2011). Pandas: A foundational Python library for data analysis and statistics. Python High Perform. Sci. Comput..

[20] Virtanen P., Gommers R., Oliphant T.E., Haberland M., Reddy T., Cournapeau D., Burovski E., Peterson P., Weckesser W., Bright J. (2020). SciPy 1.0: Fundamental algorithms for scientific computing in Python. Nat. Methods.

[21] Hunter J.D. (2007). Matplotlib: A 2D graphics environment. Comput. Sci. Eng..

[22] Lundberg S.M., Lee S.-I. A unified approach to interpreting model predictions. Proceedings of the 31st International Conference on Neural Information Processing Systems.

[23] Olson R.S., Moore J.H. TPOT: A tree-based pipeline optimization tool for automating machine learning. Proceedings of the Workshop on Automatic Machine Learning.

[24] Moez A. PyCaret: An Open Source, Low-Code Machine Learning Library in Python. https://www.pycaret.org.

[25] Little R.J. (1988). A test of missing completely at random for multivariate data with missing values. J. Am. Stat. Assoc..

[26] Van Buuren S., Groothuis-Oudshoorn K. (2011). mice: Multivariate imputation by chained equations in R. J. Stat. Softw..

[27] R Core Team (2013). R: A Language and Environment for Statistical Computing.

[28] What’s Considered a Good Log Loss?. https://stats.stackexchange.com/questions/276067/whats-considered-a-good-log-loss.

[29] Kerr K.F., Wang Z., Janes H., McClelland R.L., Psaty B.M., Pepe M.S. (2014). Net reclassification indices for evaluating risk-prediction instruments: A critical review. Epidemiology.

[30] Dumas F., Cariou A., Manzo-Silberman S., Grimaldi D., Vivien B., Rosencher J., Empana J.-P., Carli P., Mira J.-P., Jouven X. (2010). Immediate percutaneous coronary intervention is associated with better survival after out-of-hospital cardiac arrest: Insights from the PROCAT (Parisian Region Out of hospital Cardiac ArresT) registry. Circ. Cardiovasc. Interv..

[31] Kern K.B., Lotun K., Patel N., Mooney M.R., Hollenbeck R.D., McPherson J.A., McMullan P.W., Unger B., Hsu C.-H., Seder D.B. (2015). Outcomes of comatose cardiac arrest survivors with and without ST-segment elevation myocardial infarction: Importance of coronary angiography. JACC Cardiovasc. Interv..

[32] Garcia S., Drexel T., Bekwelem W., Raveendran G., Caldwell E., Hodgson L., Wang Q., Adabag S., Mahoney B., Frascone R. (2016). Early access to the cardiac catheterization laboratory for patients resuscitated from cardiac arrest due to a shockable rhythm: The Minnesota Resuscitation Consortium Twin Cities Unified Protocol. J. Am. Heart Assoc..

[33] Patel N., Patel N.J., Macon C.J., Thakkar B., Desai M., Rengifo-Moreno P., Alfonso C.E., Myerburg R.J., Bhatt D.L., Cohen M.G. (2016). Trends and outcomes of coronary angiography and percutaneous coronary intervention after out-of-hospital cardiac arrest associated with ventricular fibrillation or pulseless ventricular tachycardia. JAMA Cardiol..

[34] Kim Y.-J., Kim Y.H., Lee B.K., Park Y.S., Sim M.S., Kim S.J., Oh S.H., Lee D.H., Kim W.Y. (2019). Immediate versus early coronary angiography with targeted temperature management in out-of-hospital cardiac arrest survivors without ST-segment elevation: A propensity score-matched analysis from a multicenter registry. Resuscitation.

[35] Waldo S.W., Chang L., Strom J.B., O’Brien C., Pomerantsev E., Yeh R.W. (2015). Predicting the presence of an acute coronary lesion among patients resuscitated from cardiac arrest. Circ. Cardiovasc. Interv..

[36] Harhash A.A., May T.L., Hsu C.-H., Agarwal S., Seder D.B., Mooney M.R., Patel N., McPherson J., McMullan P., Riker R. (2021). Risk stratification among survivors of cardiac arrest considered for coronary angiography. J. Am. Coll. Cardiol..

[37] Hosmane V.R., Mustafa N.G., Reddy V.K., Reese C.L., DiSabatino A., Kolm P., Hopkins J.T., Weintraub W.S., Rahman E. (2009). Survival and neurologic recovery in patients with ST-segment elevation myocardial infarction resuscitated from cardiac arrest. J. Am. Coll. Cardiol..

[38] Khan M.S., Shah S.M.M., Mubashir A., Khan A.R., Fatima K., Schenone A.L., Khosa F., Samady H., Menon V. (2017). Early coronary angiography in patients resuscitated from out of hospital cardiac arrest without ST-segment elevation: A systematic review and meta-analysis. Resuscitation.

[39] Callaway C.W., Donnino M.W., Fink E.L., Geocadin R.G., Golan E., Kern K.B., Leary M., Meurer W.J., Peberdy M.A., Thompson T.M. (2015). Part 8: Post–cardiac arrest care: 2015 American Heart Association guidelines update for cardiopulmonary resuscitation and emergency cardiovascular care. Circulation.

[40] Lemkes J.S., Janssens G.N., van der Hoeven N.W., Jewbali L.S.D., Dubois E.A., Meuwissen M., Rijpstra T.A., Bosker H.A., Blans M.J., Bleeker G.B. (2019). Coronary Angiography after Cardiac Arrest without ST-Segment Elevation. N. Engl. J. Med..

[41] Desch S., Freund A., Akin I., Behnes M., Preusch M.R., Zelniker T.A., Skurk C., Landmesser U., Graf T., Eitel I. (2021). Angiography after Out-of-Hospital Cardiac Arrest without ST-Segment Elevation. N. Engl. J. Med..

[42] Bascom K.E., Dziodzio J., Vasaiwala S., Mooney M., Patel N., McPherson J., McMullan P., Unger B., Nielsen N., Friberg H. (2018). Derivation and validation of the CREST model for very early prediction of circulatory etiology death in patients without ST-segment–elevation myocardial infarction after cardiac arrest. Circulation.

[43] Rab T., Kern K.B., Tamis-Holland J.E., Henry T.D., McDaniel M., Dickert N.W., Cigarroa J.E., Keadey M., Ramee S., Interventional Council, American College of Cardiology (2015). Cardiac arrest: A treatment algorithm for emergent invasive cardiac procedures in the resuscitated comatose patient. J. Am. Coll. Cardiol..

[44] Noh Y.-K., Park J.Y., Choi B.G., Kim K.-E., Rha S.-W. (2019). A machine learning-based approach for the prediction of acute coronary syndrome requiring revascularization. J. Med. Syst..

[45] Zheng A., Casari A. (2018). Feature Engineering for Machine Learning: Principles and Techniques for Data Scientists.

