# Supplementary material for: Early Identification of Resuscitated Patients with a Significant Coronary Disease in Out-of-Hospital Cardiac Arrest Survivors without ST-Segment Elevation
Source: J Clin Med. 2021 Dec 2;10(23):5688. doi: 10.3390/jcm10235688 (PMC8658463; doi:10.3390/jcm10235688)
Supplement: Supplementary file 1 [file jcm-10-05688-s001.zip › Table S1.pdf]

Table S1. The definition and collection form of the variables in Korean Hypothermia Network prospective registry (KORHN-PRO).

Variables	Definition	Collection form
Age	Based on the date of birth. If the date of birth is not known, record estimated age	years
Gender	sex	Male/female
Comorbid disease	The patient has a documented history of other disease conditions that existed before the cardiac arrest.	
Previous Arrest	Previous history of cardiac arrest	Yes/No/Unknown
Previous acute myocardial infarction	Previous history of acute myocardial infarction	Yes/No/Unknown
Previous angina	Previous history of angina	Yes/No/Unknown
Previous arrhythmia	Previous history of cardiac arrhythmia	Yes/No/Unknown
Chronic heart failure	Previous history of chronic heart failure	Yes/No/Unknown
Previous TIA or stroke	Previous history of transient ischemic attack of stroke	Yes/No/Unknown
Hypertension	Previous history of hypertension	Yes/No/Unknown
Diabetes mellitus	Previous history of diabetes mellitus	Yes/No/Unknown
Pulmonary disease	Previous history of pulmonary disease such as asthma, COPD.	Yes/No/Unknown
Neurologic disease other than CVA	Previous history of neurologic disease other than CVA such as epilepsy	Yes/No/Unknown
Chronic kidney disease	Previous history of chronic kidney disease	Yes/No/Unknown
Malignancy	Previous history of malignancy	Yes/No/Unknown
Previous PCI	Previous history of PCI	Yes/No/Unknown
Previous CABG	Previous history of CABG	Yes/No/Unknown
Previous ischemic heart disease	Previous history of AMI, PCI or CABG	Yes/No/Unknown
Family history	Medical history of family	Yes/No/Unknown
Cardiac arrest	Family history of previous cardiac arrest	Yes/No/Unknown
Angina or AMI	Family history of previous angina or AMI	Yes/No/Unknown
CVA	Family history of previous CVA	Yes/No/Unknown
Arrhythmia	Family history of previous arrhythmia	Yes/No/Unknown
Unknown	Unknown of family medical history	Yes/No/Unknown
Arrest characteristics		
Initial shockable rhythm	The initial rhythm is defined as the first cardiac rhythm present when the monitor or defibrillator is attached to the patient after a cardiac arrest.<br><br>Initial shockable rhythm is defined as ventricular fibrillation or pulseless ventricular tachycardia for the initial rhythm.<br><br>The Unknown shockable is defined when shock was performed in an AED where ECG was not confirmed<br><br>The Unknown non-shockable is defined when shock was not performed in an AED where ECG was not confirmed	1. VF<br>2. Pulseless VT<br>3. PEA<br>4. Asystole<br>5. Unknown shockable<br>6. Unknown non-shockable<br>7. Unknown
Witnessed	Defined as a cardiac arrest that is seen or heard by another person or is monitored. EMS personnel respond to a medical emergency in an official capacity as part of an organized medical response team. Bystanders are all other groups including physicians, nurses, or paramedics who witness a cardiac arrest and initiate CPR but are not part of the	1. Bystander witnessed<br>2. EMS witnessed<br>3. Unwitnessed<br>4. Unknown

	organized rescue team	
Bystander CPR	Defined as CPR performed by a person who is not responding as part of an organized emergency response system to a cardiac arrest. Physicians, nurses, and paramedics may be described as performing bystander CPR if they are not part of the emergency response system involved in the victim's resuscitation.	Yes<br>No<br>Unknown
Total collapse duration	Time from collapse to return of spontaneous circulation (ROSC).<br>ROSC is defined according to a clinical assessment that shows signs of life comprising a palpable pulse or generating a blood pressure	Minutes
Total doses of epinephrine	Amount of epinephrine administered during CPR	Milligram
Immediate ECG findings	Findings from the ECG recorded for the first time after ROSC. However, do not record reciprocal change. Choose from the following 6 items. Multiple items can be selected.	1. STEMI<br>2. ST depression<br>3. LBBB<br>4. RBBB<br>5. Non-specific ST or T patterns<br>6. Normal ST segment and T wave
Immediate laboratory findings	The first test value after ROSC	
Artery blood gas analysis, pH	The first pH of blood gas analysis from arterial blood after ROSC	pH
Lactate	The first lactate value after ROSC	mmol/L
Glucose	The first glucose value after ROSC	mg/dL
Creatinine	The first creatinine value after ROSC	mg/dL
HbA1c	The first HbAic value after ROSC	%
Extracorporeal life support	Whether ECMO was applied during hospitalization. When circulation using a pump is started before ROSC, it is defined as extracorporeal CPR, and when circulation is started after ROSC, it is defined as venous-arterial ECMO.	Yes/No

AED, automated external defibrillator; AMI, acute myocardial infarction; CABG, coronary artery bypass grafting; COPD, chronic obstructive pulmonary disease; CPR, cardiopulmonary resuscitation; CVA, Cerebrovascular accident; ECG, electrocardiogram; ECMO, extracorporeal membrane oxygenation; EMS, emergency medical services; HbA1c, glycated hemoglobin; LBBB, left bundle branch block; PCI, percutaneous coronary intervention; PEA, pulseless electrical activity; RBBB, right bundle branch block; ROSC, return of spontaneous circulation; STEMI, ST segment elevation myocardial infarction; TIA, Transient ischemic attack; VF, ventricular fibrillation; VT, ventricular tachycardia.
