# Supplementary figures and images for: Early Identification of Resuscitated Patients with a Significant Coronary Disease in Out-of-Hospital Cardiac Arrest Survivors without ST-Segment Elevation
Source: J Clin Med. 2021 Dec 2;10(23):5688. doi: 10.3390/jcm10235688 (PMC8658463; doi:10.3390/jcm10235688)

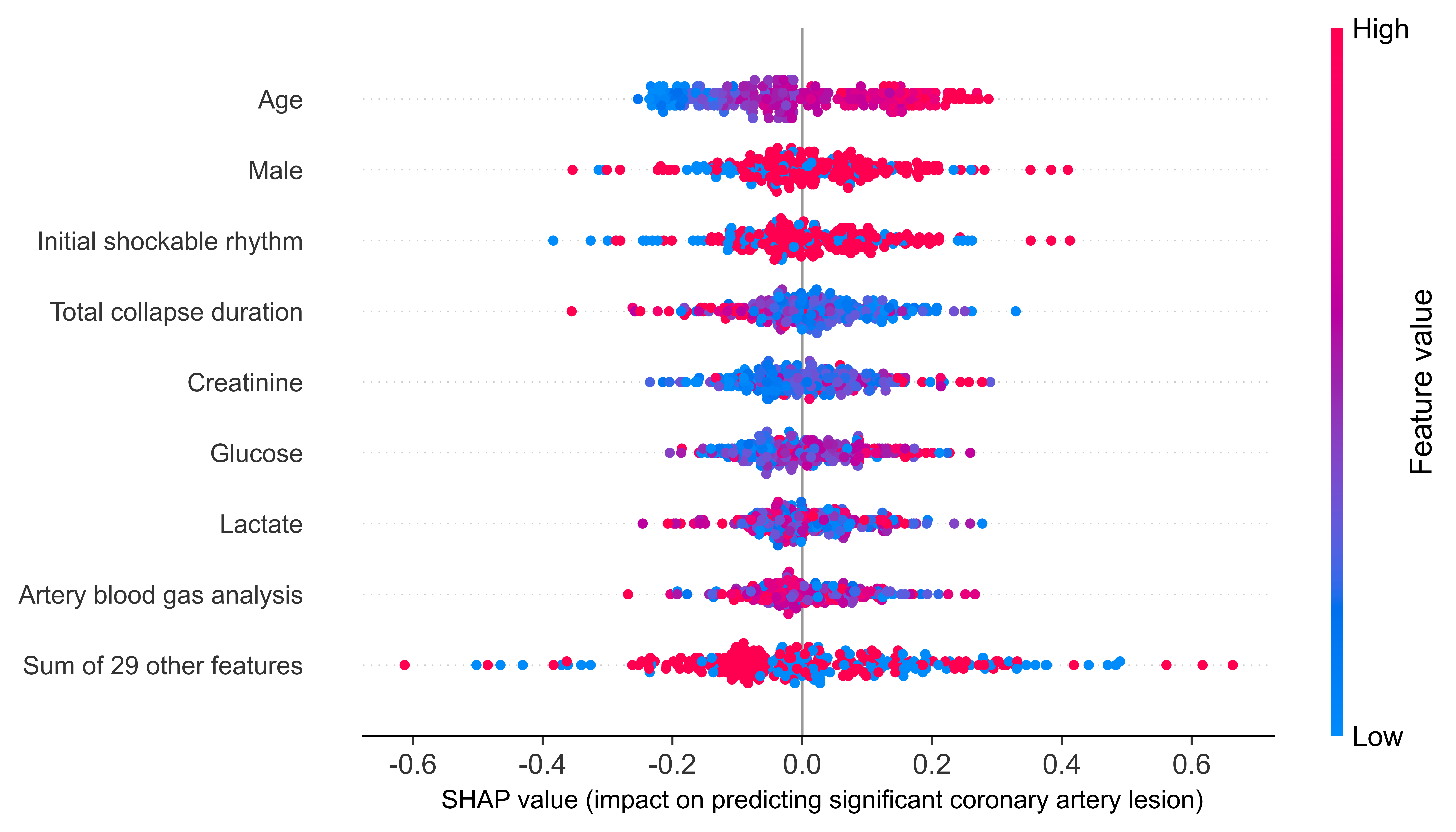

Supplement: Supplementary file 1 [file jcm-10-05688-s001.zip › Fig S3.tif]
